# Corner stores as community hubs: a systematic review of public health, economic impact, and social dynamics in urban areas

**DOI:** 10.3389/fnut.2025.1526594

**Published:** 2025-04-23

**Authors:** Hernán Darío Cadavid-Gómez, Jose Alejandro Cano, Javier A. Sánchez-Torres

**Affiliations:** ^1^Faculty of Economics and Business Management, University of Medellín, Medellín, Colombia; ^2^Business School, Universitat de Barcelona, Barcelona, Spain

**Keywords:** corner stores, neighborhood stores, systematic review, small retail business, community socioeconomics, urban food environment, socio-cultural dynamics

## Abstract

Corner stores, or neighborhood stores, are small retail spaces prevalent in urban and underserved areas, where they play a critical role in providing convenient access to food, beverages, and other necessities. This systematic review examines the multifaceted role of corner stores, focusing on their impact on public health, community dynamics, and local economies. Through an analysis of key studies, this article explores public health initiatives aimed at improving the nutritional offerings in corner stores, the socio-cultural role these stores play within communities, consumer behavior and purchasing patterns, and the unique business models that sustain them. The findings highlight the potential of corner stores to act as accessible health intervention points, culturally significant spaces, and small but impactful economic drivers in underserved neighborhoods. Despite the potential of corner stores to act as accessible intervention points for healthier eating and community development, challenges such as limited infrastructure and competition from larger retailers remain. Additionally, this systematic review highlights gaps in existing research and suggests future studies that explore long-term public health impacts, economic sustainability, and policy implications to enhance the positive contributions of corner stores to urban communities.

## 1 Introduction

Corner stores, also known as neighborhood stores, are small retail outlets commonly found in urban areas, particularly in low-income neighborhoods (1, 2). These stores play a key role in the local economy and community, providing convenient access to various goods and services (3). They often serve as convenient sources of goods and are preferred by local residents due to their proximity and ease of access, especially in neighborhoods where transportation to larger supermarkets is limited (4, 5).

Many corner stores have closed-store layouts, limited shelf space and infrastructure to stocking a wider variety of products, which limit accessibility to foods and reduce the variety of items that can be displayed, focusing on items that can be quickly and easily purchased (6). Beverages are the most frequently purchased items, with sugar-sweetened soda being particularly popular (1, 7). High-energy, low-nutrition foods such as chips, cereals, candy, pastries and canned goods are commonly found and frequently sold in nearly all stores (1, 7, 8). Likewise, tobacco products are a significant part of corner store sales (9), and hot food items and sandwiches are also among the best-selling products (7), while fruits and vegetables are often perceived as unmarketable (4). Other products that can be found in corner stores include liquor and lottery tickets, and services such as check-cashing (10).

In addition to the supply of products and services, consumers tend to visit corner stores due to factors such as convenient location, comfort, cleanliness and positive customer service (7, 11). Then, corner stores can act as community hubs, providing not just goods but also social interaction and support, often serving as a meeting point involved in community activities where residents can meet, share news, and participate in local activities, thus, maintaining the social fabric of the community, especially in areas where traditional urban centers are declining (2, 12). Consequently, these stores can help identify and prioritize community needs to engage with the local community to build a loyal customer base and contribute to the overall well-being and cohesion of the community (13, 14).

However, corner stores face several significant challenges when competing with larger retailers. Large retailers benefit from economies of scale and trade allowances from manufacturers, allowing them to lower their costs and offer competitive prices, or even engaging in aggressive pricing strategies, including loss leading, where they price some products below cost to attract customers, which puts smaller stores at a disadvantage as they cannot achieve similar cost efficiencies (15, 16). Moreover, large retailers offer a vast range of products, which attracts a significant market share that prefer the convenience of one-stop shopping (17). Additionally, large retailers are opening smaller, more centrally located stores that directly competed with independent retailers, accelerating their decline (18). Despite these challenges, some small retailers manage to survive by focusing on niche markets and offering superior customer service, which large retailers often cannot match due to their size (19).

In this regard, corner stores differentiate themselves from larger retailers and attract customers by dividing their customer base into different value segments, offering high-quality and personalized customer service, and tailoring marketing strategies to optimize their investment and enhance customer loyalty (19, 20). Another strategy of the corner stores is offering unique products that are not available at larger retailers, thereby creating a niche market (21), and developing and promoting private label products to build a unique brand image and attract a specific customer demographic (22). Enhancing the shopping experience through improved store layout, cleanliness, and customer service can significantly influence purchasing behavior (23). Additionally, the implementation of self-service technologies and QR codes can further enhance convenience and efficiency for shoppers. In this way, corner stores can create a distinct identity and attract customers who value personalized service, rewarding repeat customers and encouraging frequent visits (24).

In addition, corner stores are not only economic units through which the goods and services demanded by the community circulate, but they are also social institutions representing the inhabitants of the neighborhood, and of those who come in one way or another in search of their own present and future well-being (25). These stores actively engage with their communities through various activities such as outreach, building relationships through customer relations, giving back, partnering with community coalitions, and promoting community representation and inclusiveness (26). As a result, many customers prefer to shop at corner stores because of their proximity and ease of access, their role as centers where community members interact and where shopping experience becomes a substitute for leisure activities (27). This includes the pleasant shopping experience and good relationships with shopkeepers, which in turn influence purchase intention more than product assortment and shop design (4, 7, 28, 29).

Considering the important role that corner stores play in communities, particularly in low-income areas, there is a need to examine the existing literature on this topic. Although these stores provide critical services, they also face challenges that impact their sustainability and effectiveness as community resources (1, 2, 4, 11). A systematic review on corner and neighborhood stores is essential to understand not only their economic and social functions but also their impact on public health, consumer behavior, and community development. Several systematic reviews have examined the role of corner stores in shaping the food environment, access to healthy foods and their impact on public health. Caspi et al. (30) explored the relationship between food environments and diet, highlighting the need for rigorous spatial and store audit measures to better assess accessibility, affordability and acceptability. Glanz et al. (31) synthesized research on in-store food marketing and its influence on purchasing behavior, identifying strategies to promote healthier diets through availability and pricing interventions, while Gittelsohn et al. (32) and Langellier et al. (33) highlighted intervention strategies such as stocking healthier foods and community engagement to improve dietary behaviors.

Adam and Jensen (34) reviewed the effectiveness of food store interventions to promote healthy eating and found that most relied on increasing availability and awareness, with fewer using price incentives. Pinard et al. (35) compared small food stores in rural and urban areas, showing that rural stores lacked healthy options due to storeowner perceptions and distribution challenges, while urban stores faced transportation and safety concerns. Additionally, Kopetsky et al. (36) found that frequent food shopping was positively associated with higher fruit and vegetable consumption, suggesting potential benefits for public health interventions. More recent reviews, including Houghtaling et al. (37) and Odoms-Young et al. (38), have focused on the impact of stocking and marketing practices on health inequalities and food insecurity. While previous reviews have evaluated interventions and retail strategies in small food stores, gaps remain in understanding corner stores as multifunctional urban institutions that influence public health, economic activity and social cohesion. This study aims to bridge these perspectives by systematically analyzing the literature on corner stores, examining their role in urban environments through a multidisciplinary lens. Specifically, the study aims to answer the following research questions:

*RQ1*: How do corner stores influence public health and nutrition access in urban communities?

*RQ2*: What is the socio-economic and cultural role of corner stores within neighborhoods, particularly in low-income and minority communities?

*RQ3*: What are the predominant consumer behaviors and purchasing patterns observed in corner stores?

*RQ4*: How do business models and economic factors impact the sustainability of corner stores in competitive urban markets?

Therefore, this study aims to provide a holistic perspective on the multifaceted role of corner stores, highlighting gaps in the current research and offering recommendations for future studies. The remainder of this article is structured as follows: Section 2 presents the methodology proposed for the systematic review on corner stores. Section 3 presents the results of the bibliometric analysis, while Section 4 presents the main findings and content analysis of the most relevant documents on corner stores. Section 5 focuses on the discussion, and finally, Section 6 concludes the article and presents future lines of research.

## 2 Methodology

The purpose of this systematic review is to examine existing research on corner stores, synthesize findings across diverse studies and identify key themes and trends related to their economic, social, and health impacts within urban communities. By analyzing the existing body of literature, this paper seeks to uncover gaps in current research and offer insights that could inform policy recommendations and future research directions regarding the optimization of corner stores for community well-being. This systematic review employs a comprehensive data collection process, utilizing databases such as Scopus and Web of Science, two well-established and widely respected sources for academic literature (39). Both databases were selected for their extensive coverage of interdisciplinary studies relevant to a wide range of perspectives on corner stores could be captured.

The data collection process was structured according to the PRISMA (Preferred Reporting Items for Systematic Reviews and Meta-Analyses) guidelines (40). No additional methodological frameworks were used to guide the search strategy, as PRISMA ensures rigor and reproducibility in systematic reviews across disciplines. This framework provides a systematic approach for identifying, screening, and including relevant literature, which helps enhance the transparency and rigor of the study. Using PRISMA, an initial search was conducted in Scopus and Web of Science (WoS) with specific keywords such as “corner stores,” “neighborhood stores,” “corner shops,” and “neighborhood shops.” The search equation TITLE (“corner store” OR “neighbo* store” OR “neighbo* shop” OR “corner shop”) was used in both Scopus and WoS in mid-October 2024, yielding 121 papers in Scopus and 57 papers in WoS.

Systematic reviews in public health and social sciences often rely on title-based or title-and-abstract-based screening methods to refine large datasets and maintain specificity. Several studies, including Adam and Jensen (34), Langellier et al. (33), Pinard et al. (35), Houghtaling et al. (37), Caspi et al. (30), and Xin et al. (41), describe screening strategies where titles and abstracts are reviewed before full-text selection. This approach prioritizes relevance while reducing the risk of including studies that only tangentially mention the topic of interest. The title-based search strategy was designed to ensure that the retrieved studies explicitly focused on corner stores as a primary research subject, enhancing thematic consistency and methodological rigor.

**Figure 1:**
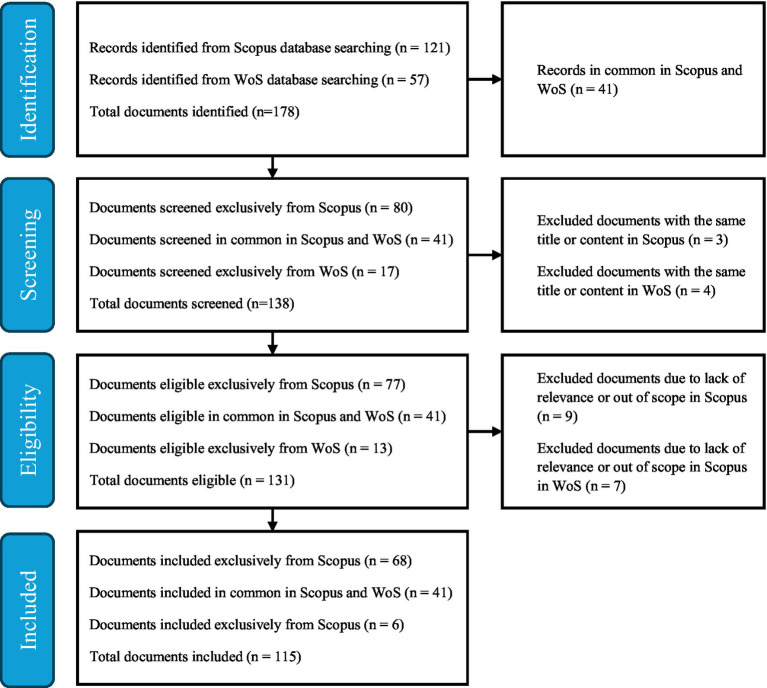
PRIMSA flow diagram for document inclusion.

As shown in Figure 1, in the identification stage, 41 documents were found to be common between Scopus and WoS, resulting in 138 unique documents between the two databases. Then, in the screening stage, three documents from Scopus and four from WoS were excluded because they had the same title or content, resulting in 138 unique documents. In the eligibility stage, nine documents from Scopus and seven documents from WoS were excluded because they dealt with topics not relevant to corner stores. These excluded papers deal with topics related to poetry, the airline industry, cigarette consumption in different scenarios, the approach of large corporations, language and semantics, archival analysis of corporate history, historical narrative, interview of a book on environmental protection, among others. Therefore, articles were then included for relevance, with inclusion criteria focusing on studies that addressed the economic, social, or health impacts of corner stores within communities, leaving a refined set of 115 documents that forms the basis of this systematic review.

The study integrates both qualitative and quantitative data collection methods. The quantitative component is based on a bibliometric analysis conducted on the 115 documents from Scopus and Web of Science. This quantitative method includes examining metrics such as publication frequency, citation counts, the distribution of research across different journals and disciplines, author collaboration, publications by territory, affiliations, and keywords, highlighting trends over time and identifying influential studies, authors, and institutions within the field (42). The bibliometric analysis also uses VOSviewer software to generate co-authorship and co-occurrence analyses. For the co-authorship analysis, the full count method was used, allowing a minimum of five documents per author, resulting in 12 authors representing those with the highest total link strength. For the co-occurrence analysis, the full count method was also used, taking author keywords as the unit of analysis and considering a minimum of five occurrences for a keyword, resulting in 14 keywords that met the threshold. The selected keywords represent those with the highest total link strength.

On the other hand, the qualitative component involves a content analysis of the selected articles that enables the identification of recurring themes, patterns, and perspectives related to corner stores. Through content analysis, key concepts and ideas are extracted from the literature, allowing for a narrative synthesis that explains the multifaceted impact of corner stores. By combining content analysis and bibliometric analysis, this study ensures a comprehensive review of the literature, blending descriptive insights with quantitative metrics to address research topics around corner stores.

## 3 Bibliometric analysis

### 3.1 Publication patterns and research focus

**Figure 2:**
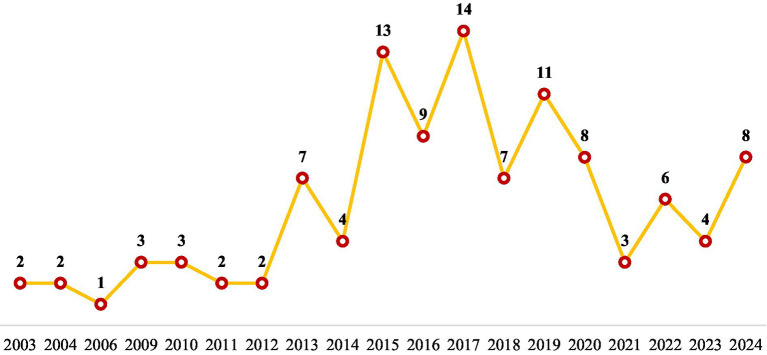
Documents published by year.

Based on the 115 documents identified with the PRISMA guidelines, Figure 2 shows that research on corner stores has expanded significantly since 2013, with notable peaks in 2015, 2017, and 2019. Table 1 shows that the primary subject areas are medicine (30.3%), social sciences (21.2%), and nursing (10.4%), which highlights the strong focus on public health and nutrition. Other fields such as business, environmental science, and economics contribute insights into retail operations and urban development.

**Table 1:** Subject areas for corner stores.

Subject area	% Ind.	% Accum.
Medicine	30.3%	30.3%
Social Sciences	21.2%	51.5%
Nursing	10.4%	61.9%
Business, Management and Accounting	6.9%	68.8%
Environmental Science	5.6%	74.5%
Agricultural and Biological Sciences	5.2%	79.7%
Arts and Humanities	4.8%	84.4%
Economics, Econometrics and Finance	2.6%	87.0%
Computer Science	2.2%	89.2%
Engineering	2.2%	91.3%
Others (11 subject areas)	8.7%	100.0%

Table 2 illustrates that most publications (80.9%) are journal articles, demonstrating their scholarly rigor, while book chapters (7.8%) and conference papers represent a smaller portion. Access remains a challenge, as only 49.6% of studies offer open access, limiting availability to researchers and policymakers. English dominates (97.4%), ensuring global reach, but a broader linguistic diversity could enhance inclusivity.

**Table 2:** Document type for literature about corner stores.

Document type	Documents	% Ind.
Article	93	80.9%
Book chapter	9	7.8%
Meeting Abstract	3	2.6%
Editorial	2	1.7%
Article; Book Chapter	2	1.7%
Note	2	1.7%
Short survey	1	0.9%
Conference paper	1	0.9%
Report	1	0.9%
Review	1	0.9%

### 3.2 Citations

Citation analysis highlights the most influential publications in corner store research. The most frequently cited articles explore the role of corner stores in shaping food environments and childhood obesity, underscoring their impact on dietary behaviors and public health interventions (Table 3). Several highly cited studies focus on nutrition interventions, consumer purchasing patterns, and the availability of healthy food options in low-income urban areas. Therefore, citation trends confirm that corner store research is closely tied to public health, nutrition policy, and food accessibility, reinforcing their role as critical community resources in urban environments.

**Table 3:** Most cited documents per year.

Authors	Title	Source	Year	Scopus Cites	WoS Cites	Avg. Scopus Cites	Avg. WoS Cites
Borradaile et al. (43)	Snacking in children: The role of urban corner stores	Pediatrics	2009	175	–	11.05	–
Song et al. (64)	A corner store intervention in a low-income urban community is associated with increased availability and sales of some healthy foods	Public Health Nutrition	2009	143	142	9.03	8.97
Dannefer et al. (61)	Healthy bodegas: Increasing and promoting healthy foods at corner stores in New York City	American Journal of Public Health	2012	114	-	8.89	-
Cavanaugh et al. (8)	Nutrition environments in corner stores in Philadelphia	Preventive Medicine	2013	95	–	8.03	–
Hua et al. (137)	Multilingual, multisensory and multimodal repertoires in corner shops, streets and markets: introduction	Social Semiotics	2017	60	–	7.66	–
Thorndike et al. (118)	Choice architecture to promote fruit and vegetable purchases by families participating in the Special Supplemental Program for Women, Infants, and Children (WIC): Randomized corner store pilot study	Public Health Nutrition	2017	54	53	6.90	6.77
Gittelsohn et al. (138)	Process Evaluation of Baltimore Healthy Stores: A Pilot Health Intervention Program With Supermarkets and Corner Stores in Baltimore City	Health Promotion Practice	2010	88	–	5.93	–
Lent et al. (1)	Corner store purchases made by adults, adolescents and children: Items, nutritional characteristics and amount spent	Public Health Nutrition	2015	56	53	5.70	5.39
Caspi et al. (89)	Food and beverage purchases in corner stores, gas-marts, pharmacies and dollar stores	Public Health Nutrition	2017	44	–	5.62	–
Furr-Holden et al. (139)	Not in my back yard: A comparative analysis of crime around publicly funded drug treatment centers, liquor stores, convenience stores, and corner stores in one Mid-Atlantic City	Journal of Studies on Alcohol and Drugs	2016	49	–	5.55	–
O’Malley et al. (5)	Feasibility of increasing access to healthy foods in neighborhood corner stores	Journal of Community Health	2013	61	–	5.16	–
Cavanaugh et al. (72)	Changes in food and beverage environments after an urban corner store intervention	Preventive Medicine	2014	55	51	5.08	4.71
Lucan et al. (46)	Storing empty calories and chronic disease risk: Snack-food products, nutritive content, and manufacturers in Philadelphia corner stores	Journal of Urban Health	2010	75	–	5.06	–

Of the 78 sources in which the 115 documents analyzed are published, Table 4 shows the journals with the highest impact, where the impact is measured by cumulative citations on the topic of corner stores and the H-index, which represents the number of documents that have received at least h citations. In this case, Public Health Nutrition is the journal with the highest impact on the topic of corner stores, with 396 citations in Scopus, 268 citations in WoS and an H-index of 7, which means that at least 7 articles on corner stores have received at least 7 citations each. From this list of journals, in addition to journals in the fields of public health and nutrition, journals in other fields are identified, such as the Journal of Sociolinguistics, Social Semiotics, Journal of Political Marketing and Social and Cultural Geography, which are characterized by having received between 28 and 69 cumulative citations on the topic of corner stores.

**Table 4:** Highest impact journals.

Journal	Publisher	Documents	Cites Scopus	Cites WoS	H-index	Max
Public Health Nutrition	Cambridge University Press	7	396	268	7	396
Preventive Medicine	Academic Press	3	190	91	3	190
Pediatrics	American Academy of Pediatrics	1	175	–	1	175
Health Promotion Practice	SAGE Publications Inc.	3	139	2	3	139
Journal of Community Health	Springer Netherlands0	3	135	74	2	135
American Journal of Public Health	American Public Health Association	1	114	–	1	114
Preventing Chronic Disease	Centers for Disease Control and Prevention	5	75	62	4	75
Journal of Urban Health	Springer Science and Business Media Deutschland GmbH	1	75	–	1	75
Journal of Sociolinguistics	Blackwell Publishing Ltd	1	69	48	1	69
International Journal of Environmental Research and Public Health	MDPI AG	5	66	17	5	66
Social Semiotics	Routledge	1	60	–	1	60
Journal of Studies on Alcohol and Drugs	Alcohol Research Documentation Inc.	1	49	–	1	49
BMC Public Health	BioMed Central Ltd.	1	41	39	1	41
Obesity	Blackwell Publishing Inc.	1	38	36	1	38
Family and Community Health	Lippincott Williams and Wilkins Ltd	1	33	30	1	33
Preventive Medicine Reports	Elsevier Inc.	2	33	7	2	33
Journal of Political Marketing	Routledge	1	32	–	1	32
Frontiers in Public Health	Frontiers Media S. A	1	31	–	1	31
Social and Cultural Geography	Routledge	1	–	28	1	28
Journal of Hunger and Environmental Nutrition	Taylor and Francis Ltd.	7	26	–	3	26

### 3.3 Authorship

Collaboration is high, with 75.7% of publications co-authored. The collaboration index (5.85) reflects strong teamwork, particularly in public health. Table 5 shows that key contributors include Joel Gittelsohn (Johns Hopkins Bloomberg School of Public Health), Allison Karpyn (University of Delaware), and Sandy Sherman (The Food Trust), who have authored multiple influential studies.

**Table 5:** Authors with the highest scientific output.

Authors	Affiliation	Country	Databases			Total Docs.
			Scopus	WoS	Scopus and WoS	
Gittelsohn, Joel	Johns Hopkins Bloomberg School of Public Health	United States	Gittelsohn et al. (138), Gudzune et al. (124), Mui et al. (66), Nau et al. (100), Ross et al. (71), Wensel et al. (69), Lewis et al. (130)	Liu et al. (140)	Song et al. (64), Lewis et al. (94)	10
Karpyn, Allison E.	University of Delaware	United States	Borradaile et al. (43), Lucan et al. (46), DeWeese et al. (141)	DeWeese et al. (142)	Mayer et al. (2)	6
Sherman, Sandy	The Food Trust	United States	Borradaile et al. (43), Lucan et al. (46), Sherman et al. (47)	–	Lent et al. (1, 45), Lawman et al. (44)	6
Vander Veur, Stephanie S.	Temple University	United States	Borradaile et al. (43), Sherman et al. (47), Lawman et al. (9)	–	Lent et al. (1, 45), Lawman et al. (44)	6
Foster, Gary D.	University of Pennsylvania Perelman School of Medicine	United States	Borradaile et al. (43), Sherman et al. (47), Lawman et al. (9)	–	Lent et al. (1, 45), Lawman et al. (44)	6
Prelip, Michael L.	UCLA Fielding School of Public Health	United States	Sharif et al. (11, 51)	–	Ortega et al. (49, 50), Albert et al. (48)	5
Glik, Deborah Carrow	UCLA Fielding School of Public Health	United States	Sharif et al. (11, 51)	–	Ortega et al. (49, 50), Albert et al. (48)	5
Sharif, Mienah Zulfacar	University of Washington	United States	Sharif et al. (11, 51)		Ortega et al. (49, 50), Albert et al. (48)	5
Ortega, Alexander N.	University of Hawaiʻi at Mānoa	United States	Sharif et al. (11, 51)	–	Ortega et al. (49, 50), Albert et al. (48)	5
Mallya, Giridhar G.	Robert Wood Johnson Foundation	United States	Cavanaugh et al. (8), Lawman et al. (9)	–	Cavanaugh et al. (72), Lawman et al. (44), Lent et al. (1)	5
Sandoval, Brianna Almaguer	The Food Trust	United States	Borradaile et al. (43), Sherman et al. (47)	–	Lent et al. (1, 45), Lawman et al. (44)	5
McCoy, Tara Alexis	Temple University	United States	Borradaile et al. (43), Sherman et al. (47)	–	Lent et al. (1, 45), Lawman et al. (44)	5
Wojtanowski, Alexis C.	WW International, Inc	United States	Sherman et al. (47), Lawman et al. (9)	–	Lent et al. (45), Lawman et al. (44)	4
Albert, Stephanie L.	NYU Grossman School of Medicine	United States	Sharif et al. (11)		Ortega et al. (49, 50), Albert et al. (48)	4
Chan-Golston, Alec M.	UC Merced	United States	Sharif et al. (11)	–	Ortega et al. (49, 50), Albert et al. (48)	4
Langellier, Brent A.	Drexel University	United States	Sharif et al. (51)	–	Ortega et al. (49, 50), Albert et al. (48)	4
Laska, Melissa N.	University of Minnesota Twin Cities	United States	Caspi et al. (89), Hearst et al. (90)		Jilcott Pitts et al. (56, 143)	4
McGuirt, Jared T.	The University of North Carolina at Greensboro	United States	–	–	Jilcott Pitts et al. (56, 143), Pitts et al. (87), Haynes-Maslow et al. (68)	4

**Figure 3:**
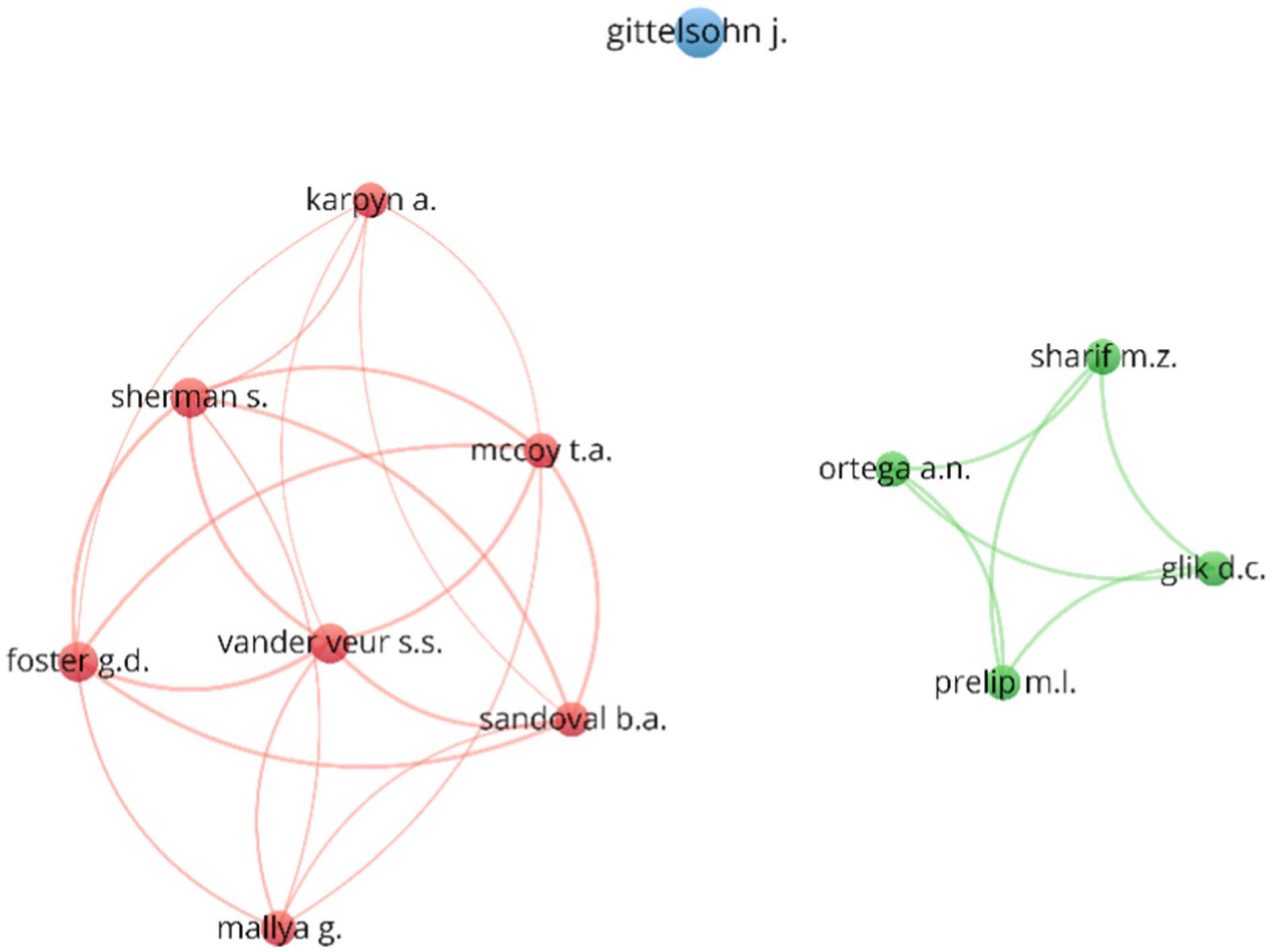
Co-authorship analysis.

From the co-authorship analysis, three co-authorship clusters are identified, as shown in Figure 3, where a first cluster highlights authors such as Karpyn, Sherman, Vander Veur, Foster, Mallya, Sandoval and McCoy, who are linked by several common articles (1, 9, 43–47). A second cluster consists of Prelip, Glik, Sharif and Ortega, who have co-authored four papers (11, 48–51). The third cluster is made up of a single author, Gittelsohn, who contributes the largest number of papers on corner stores, but his co-authors do not achieve a significant number of published papers to appear in the above cluster.

**Figure 4:**
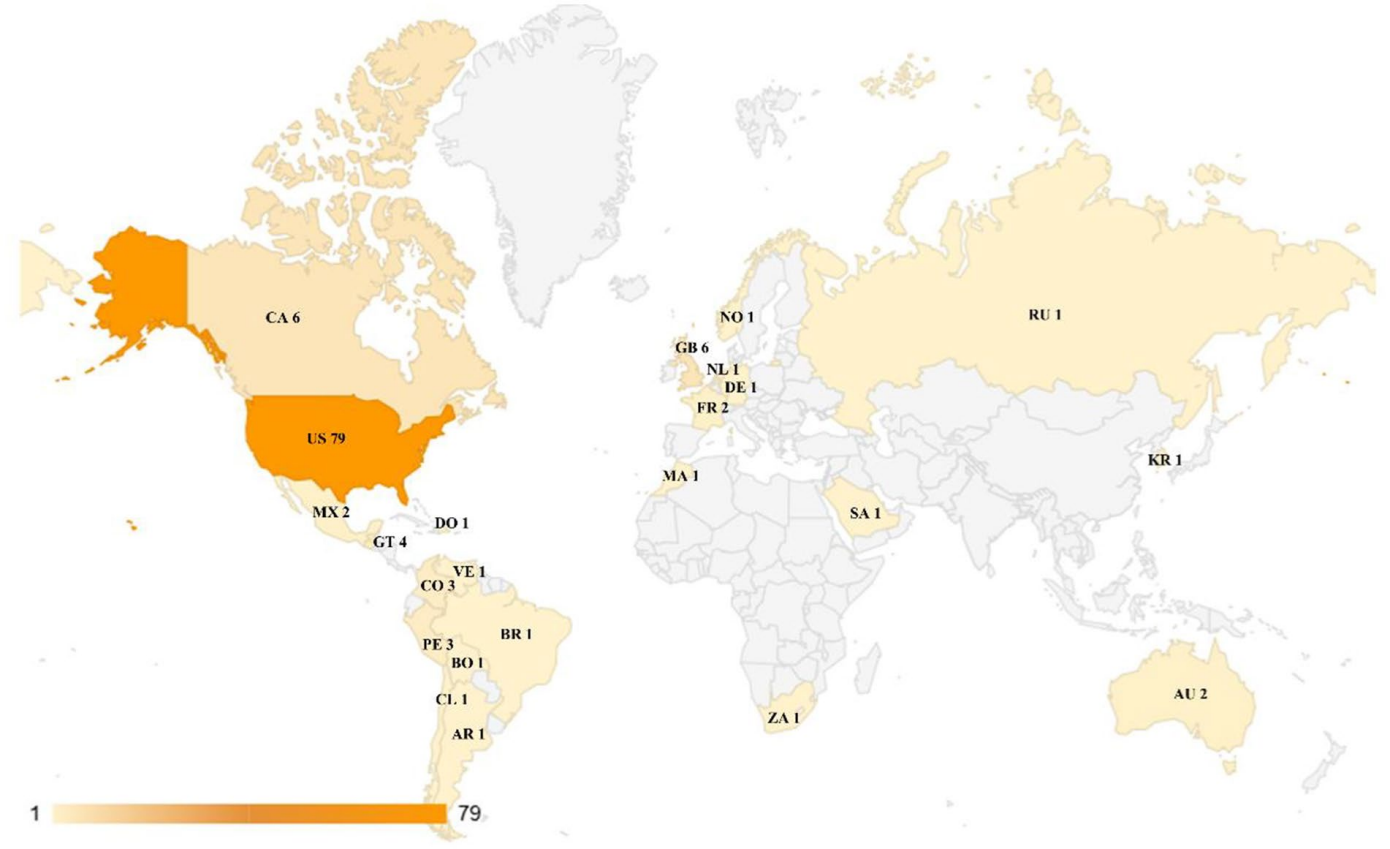
Document production by country/territory.

Regarding the territorial distribution of scientific production on corner stores, Figure 4 shows that the United States dominates research output (68.7%), followed by Canada and the UK (5.2% each). Latin America (15.7%) shows growing interest, while contributions from Asia, Africa, and Oceania remain limited. Institutional leadership (Table 6) aligns with this trend, as the top publishing institutions are primarily based in United States.

**Table 6:** Affiliations with most publications.

Affiliation	Documents	% Ind
Johns Hopkins Bloomberg School of Public Health (United States)	11	18.0%
Johns Hopkins University (United States)	8	13.1%
The Food Trust (United States)	8	13.1%
The University of North Carolina at Chapel Hill (United States)	7	11.5%
UCLA Fielding School of Public Health (United States)	6	9.8%
Philadelphia Department of Public Health (United States)	5	8.2%
University of California, Los Angeles (United States)	5	8.2%
University of Toronto (Canada)	5	8.2%
Albert Einstein College of Medicine (United States)	4	6.6%
East Carolina University (United States)	4	6.6%
Rutgers University–New Brunswick (United States)	4	6.6%
Temple University (United States)	4	6.6%
University of Delaware (United States)	4	6.6%
University of Minnesota Twin Cities (United States)	4	6.6%
University of Pennsylvania (United States)	4	6.6%
University of Pennsylvania Perelman School of Medicine (United States)	4	6.6%

### 3.4 Keywords and concepts

**Figure 5:**
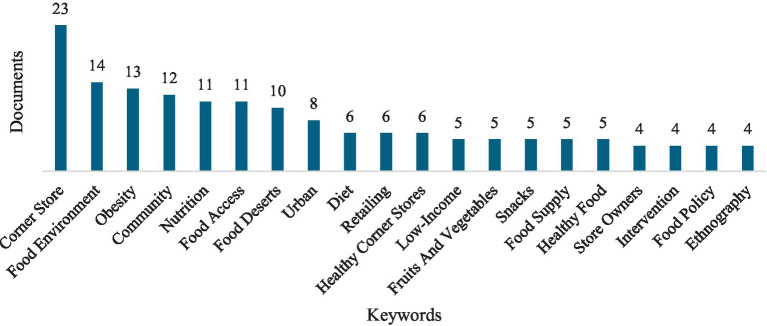
Relevant keywords for corner stores.

Keyword analysis reveals three major thematic clusters in corner store research (Figure 5). The first cluster focuses on food environments, obesity, and diet highlighting concerns about nutrition and health disparities. The second cluster emphasizes community and retailing, examining how corner stores serve as economic and social hubs in urban settings. The third cluster centers on consumer behavior and purchasing patterns, particularly in low-income neighborhoods.

**Figure 6:**
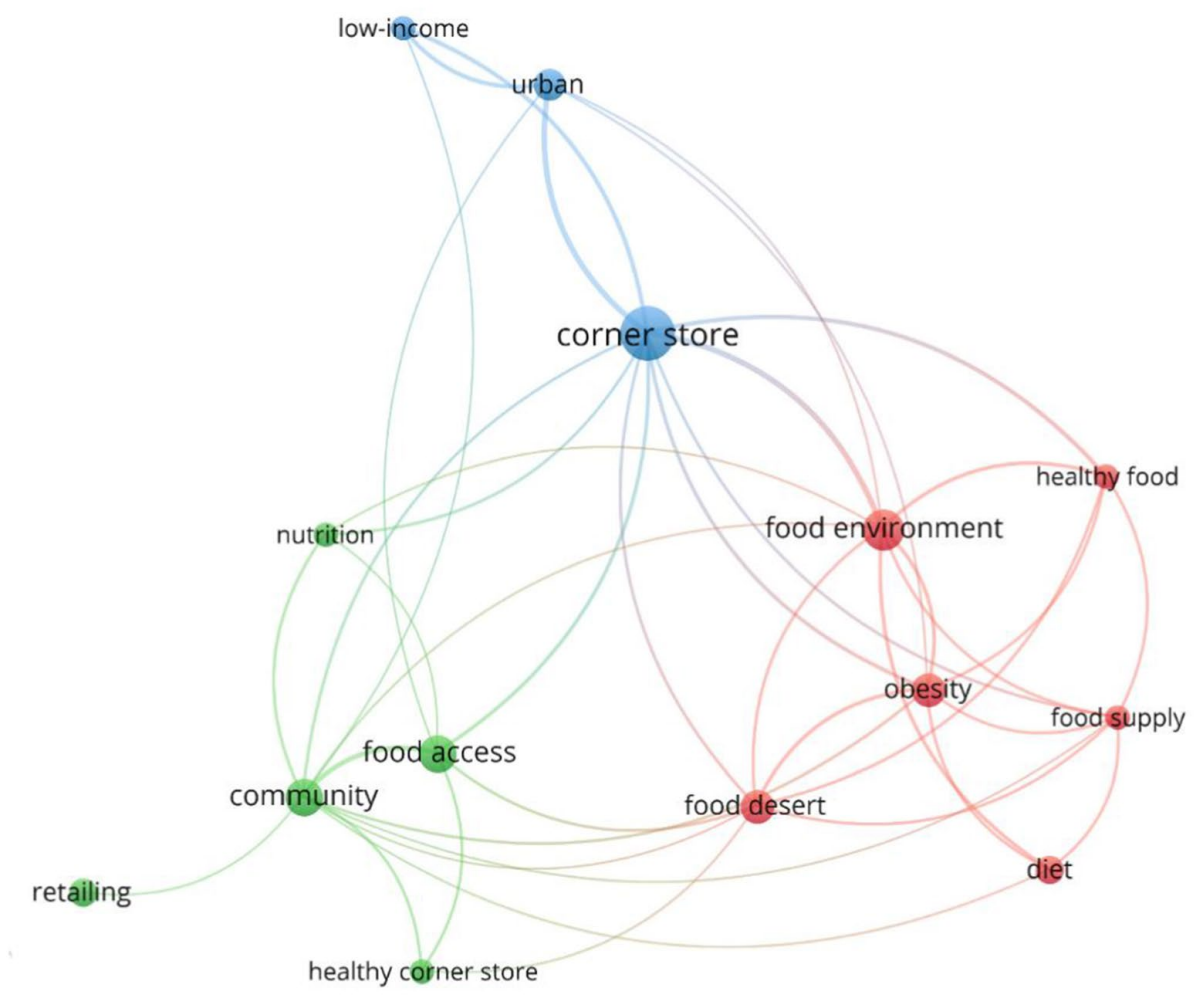
Co-occurrence analysis for keywords.

The co-occurrence analysis from Figure 6 shows strong associations between corner stores, food deserts, and healthy food access, reinforcing the role of these stores in shaping dietary choices. Additionally, Table 7 shows the keyword frequency analysis, indicating that the clusters of food environment, community, and corner store are related to dominant research topics such as public health, nutrition, and urban food systems, aligning with ongoing discussions on food accessibility and community health initiatives.

**Table 7:** Co-occurrence analysis for keywords.

Cluster	Keywords of the clusters	Occurrences	Total links strength
Cluster 1: Food environment	Food environment	13	23
	Obesity	9	18
	Food desert	9	15
	Food Supply	5	13
	Healthy food	5	13
	Diet	6	8
Cluster 2: Community	Community	11	19
	Food access	11	14
	Nutrition	5	6
	Healthy corner store	5	5
	Retailing	6	1
Cluster 3: Corner store	Corner store	23	36
	Low-income	5	9
	Urban	8	14

## 4 Findings

The bibliometric analysis highlights key trends in corner store research, revealing both strengths and gaps in the existing literature. The dominance of public health and nutrition studies suggests that corner stores are primarily examined through the lens of food access and dietary behaviors, with less emphasis on their economic and social functions. While interventions promoting healthier food availability have been widely studied, fewer works explore long-term business sustainability, consumer decision-making beyond nutrition, and the broader socio-economic role of these stores in urban communities. This gap underscores the need for interdisciplinary approaches that integrate urban economics, behavioral science, and community development to fully understand the significance of corner stores. Collaboration trends indicate that a limited number of research clusters and institutions, which are primarily in the United States and Canada, drive much of the scholarship on corner stores. The concentration of studies in high-income countries suggests a lack of research on corner stores in the Global South, where they play an even more critical role in local economies and informal food systems.

Based on the main themes suggested by the keywords found in the bibliometric analysis, a content analysis of the documents included is carried out from the point of view of themes related to corner stores, such as public health and nutrition initiatives, community, cultural and socio-economic role, consumer behavior and purchasing patterns, business models and economic impact.

### 4.1 Public health and nutrition initiatives in corner stores

According to the literature, corner stores are sometimes viewed negatively due to their association with selling unhealthy products and contributing to poor diet-related health outcomes (10, 11). As food insecurity significantly impacts overall public health (52), healthy corner store programs are devoted to provide a proper food environment and change consumer behavior (53). Corner stores are increasingly recognized as key points for nutrition interventions to influence consumers to make healthier choices more frequently (54), especially in food deserts or underserved urban areas with limited access to fresh and healthy foods (55). Several studies emphasize that improving the availability of nutritious options in these stores can positively influence dietary habits (56, 57), particularly among low-income and minority populations who rely on them as primary food sources (58, 59).

Customers, including children and adolescents, frequently purchase beverages, chips, and prepared food items, which are often high in sugar and sodium (1, 47). In this sense, Sadler and Saxe-Custack (60) indicate that corner stores can influence children’s diets, so it is necessary to examine children’s purchasing patterns before and after school in proximity to urban corner stores, since children frequently bought high-calorie, low-nutrition items like sugary snacks and drinks. However, intervention programs that included fresh produce and healthier snack alternatives were linked to moderate shifts in purchasing habits (45), highlighting the importance of creating child-targeted nutritional interventions at corner stores to reduce calorie-dense, nutrient-poor food intake and promote healthier alternatives (46). Dannefer et al. (61), Lucan et al. (46) and Albert et al. (48) demonstrate the potential for corner stores to expand access to healthier foods through targeted interventions by conducting studies on the effects of introducing healthier food items in corner stores. They observed that the increased availability of fresh produce, whole grains, and low-fat dairy products could significantly influence purchasing patterns. These initiatives faced challenges, including consumer demand for healthier items, logistical issues related to space and refrigeration, and the structure and relations of the food system (62, 63). Then, these findings suggest that the success of corner store interventions may depend on simultaneous efforts to promote awareness and affordability of healthy food options within these communities (59).

Similarly, Song et al. (64) and Young et al. (65) report on the outcomes of culturally tailored programs to increase sales, focusing on community-specific strategies, such as increasing the stock and promotion of healthy foods and engaging residents through food justice events and taste-testing demonstrations. They also found that store owners participating in these initiatives expressed a willingness to sustain the changes if given adequate support and resources. Thus, in order to improve the relationship and sourcing with suppliers that provide mainly healthy food/beverages, it is necessary to address the ethnic and cultural characteristics of the store owners (66).

Mah et al. (67) emphasizes that intersectoral collaboration between health units, local governments, and business owners is essential for sustaining corner store interventions. This approach underscores the importance of partnerships and training in maintaining a consistent stock of nutritious items and effectively promoting them to local communities. Similarly, external support, such as training for store owners, point-of-purchase promotions, and nutritional labeling, has been shown to play a crucial role in the successful adoption of healthier food stocking practices (68, 69). Specific to store owners, they face challenges such as obtaining high-quality produce at reasonable prices and in small quantities, which affects their ability to offer healthier options (2). DeWeese et al. (70) highlight the need for assisting them in understanding product properties and nutritional content, which is key to encouraging the stocking of a broader variety of fruits and vegetables. Interventions and support for store owners can also be used through a smartphone application to facilitate the distribution of healthy food (71).

Further support strategies aimed at encouraging customers to choose healthier food options, thereby improving nutrition and addressing obesity in low-income populations, include placing healthy items at checkout aisles, using in-store signage to promote these options, and enlisting community-based organizations for support. Additionally, infrastructure enhancements, such as refrigeration for low-fat milk, fruits, and vegetables, shelving enhancements and improved storage facilities, are often necessary to make these interventions feasible and sustainable (63, 72). In addition to food sales, corner stores become primary antibiotic dispensaries in countries where antibiotics such as amoxicillin and tetracycline are widely available without prescription, which can facilitate overuse and contribute to antimicrobial resistant bacteria. This represents a need to implement regulations to change the availability of antibiotics to regulate public health (73).

### 4.2 Community, cultural and socio-economic role of corner stores

The literature shows that corner stores serve as essential community institutions in urban neighborhoods, particularly within low-income and immigrant communities, where they fulfill roles beyond simple retail (74). Corner stores are not only a source of commercial products and services, but also a recreational center, a place for social gatherings and neighborhood activities, fostering relationships and social cohesion (25, 75). This social function is crucial, especially during crises like the COVID-19 pandemic, where the shopping experience becomes a substitute for leisure activities (29).

Studies have highlighted these stores as spaces for social interaction, cultural exchange, and economic support, shaping the dynamics of community life and resilience in significant ways (2). Everts (76) explored this phenomenon in an ethnographic study of immigrant-owned stores in Germany, revealing that interactions between shopkeepers and customers contributed to a sense of belonging and community identity. The shared practices within these stores, such as informal conversations and personalized service, create a familiar environment that reinforces social bonds (77). Similarly, Mayer et al. (2) found that store owners recognized their role as trusted community figures, often engaging in discussions with customers about health and other local issues. These interactions show that corner stores not only fulfill commercial needs but also provide a space where trust and rapport succeed (78, 79). Corner stores have even become a new space for political interaction, where candidates can interact and communicate with the local context, offering an alternative to top-down politics that do not engage with voters (80).

The cultural significance of corner stores is particularly notable in immigrant and ethnic neighborhoods, where they act as venues for cultural affirmation and identity (81). Andrade et al. (28) discuss how corner stores in Latin American communities operate on cultural values of trust, commitment, and loyalty, with store owners and customers developing close relationships that go beyond transactional interactions. In this regard, Dombrowski and Kelley (74) conceptualize corner stores as community-based enterprises where owners prioritize public health and community well-being, often over profit, reinforcing their role as culturally rooted establishments that cater to local social needs. Corner stores are critical for economic survival in economically marginalized communities by providing informal financial services, such as short-term credit (25). Puchalski (82) found that many low-income residents rely on corner convenience stores for short-term, interest-free credit, especially when conventional credit options are unavailable or unaffordable. This informal in-store credit system enables residents to manage financial instability and access essentials even during economic downturns. Hippert (79, 83) similarly observed that in the Dominican Republic, migrant Haitians relied on informal credit systems to maintain food security, underscoring how corner stores act as financial safety nets by facilitating resource exchange and offering flexible payment options.

While corner stores provide indispensable support, they also face socio-economic challenges that affect their operations and relations with the community. Gardner-Buckshaw (10) notes that urban corner stores face reputational and operational challenges due to the sale of items like liquor, cigarettes, and high-calorie snacks, which some view as “predatory” due to their potential to exploit vulnerable populations. These dynamics highlight a tension in the community role of corner stores, so while they meet immediate needs, they may also reinforce health and socio-economic issues. However, BeLue et al. (4) found that corner shop owners reported that tobacco products and hot food were their best selling items, while fruit and vegetables were perceived as unmarketable. This presents a challenge in matching community needs with corner shop provision and services, particularly given that store owners sometimes did not feel that they belonged to the community in which their corner shop was located.

As a potential solution to this issue, corner stores should respond to the perceptions and requirements of their customers to better meet community needs (84), offer stock items that cater to the cultural preferences of the local community and educate customers about unique and high-quality products to effectively engage with their local communities, increase patronage and customer satisfaction, promote community representation and inclusiveness, enhance shopping experience, and encourage loyalty (11, 26, 85). In this way, interaction and engagement with the community and stakeholders is required to incorporate improvement plans for corner store networks (13, 86), including collaboration to promote healthy food option to improve community health (87) and support economic and social structures within the community (88).

### 4.3 Consumer behavior and purchasing patterns

Consumer behavior in corner stores are significantly influenced by store location (89), product availability (90), and customer demographics (91), which together shape purchasing patterns across urban communities. Frequent purchases of high-calorie, low-nutrient items are common in corner stores, with studies showing that these purchases are often made on a daily or weekly basis (89). Kiszko et al. (7) found that consumers typically bought sugary beverages, snacks, and fast food items like sandwiches and chips. Lent et al. (1) observed that children, adolescents, and adults frequently purchased snacks and beverages high in sugar, sodium, and fat, contributing substantially to their daily caloric intake. Similarly, Borradaile et al. (43) identify that the most frequently purchased items in corner stores are high-energy low-nutritive foods and beverages, contributing significantly to energy intake among urban school children. These studies noted that most consumers frequented these stores due to proximity to their homes and convenient location, especially in areas with limited transportation options, highlighting that consumers prioritize location and ease of access over product variety (4, 7, 89).

Among low-income, urban youth, corner store shopping begins at an early age, often with family members, and many young people report visiting these stores independently. In this case, the primary factors driving their choice of a specific corner store are product offerings, expressing a desire for a mix of healthy and less-healthy foods in their ideal corner store (47). This is supported by the trend that young people are receptive to buying healthy food, such as wholefood snack packs on the corner (92), and that initiatives such as child-only vouchers can change young people’s consumption patterns, encouraging the consumption of healthier foods (93). In addition, the study by Lewis et al. (94) shows that people with low income and home ownership are associated with lower consumption of beverages and fiber, and lower consumption of fruit and vegetables. According to Tibbits et al. (95), while most customers felt it was easy to find healthy options at corner stores, less than half actually purchased any healthy foods, being women and Hispanic/Latino customers the most likely to buy at least one type of healthy item.

Regarding product offerings, corner store owners often stock products that are in high demand among their customers (4), and products representing high profits such as unhealthy snacks and sugary beverages (46, 96), so the availability of certain products, such as fruit and vegetables, will depend on the likelihood that customers will buy them (97). This means that increasing the variety of healthier food options for health-conscious consumers, especially in low-income areas, will depend on changing consumer habits (98, 99), change in infrastructure (5) and the cultural relevance of such products to meet the preferences of local customers (11). If so, Martin et al. (97) suggest that increasing the range of fruit and vegetables available in corner stores may increase consumption, particularly among food insecure and low-income populations at risk of health inequalities. Likewise, pricing experiments, such as increasing the price of sugar-sweetened beverages to lower the price of water could maintain profitability while promoting healthier choices (100).

On the other hand, understanding culture also has a direct impact on consumer loyalty (101), which can lead to the provision of personalized services, such as miniaturization, which introduce smaller, affordable product sizes to meet the consumer’s consumption needs and make products more accessible (25, 102), since the price by itself is significant to customer satisfaction (103). This is often the case for both food and other products, such as tobacco (cigars and cigarettes), which are sold individually and unpackaged in corner stores (9). In developing countries, Osakwe (104) shows that service quality components such as personal interaction, store reliability and physical store attributes, and customer characteristics such as relationship length, household size, gender and income level are necessary conditions for increasing customer share of wallet in the context of corner stores. Furthermore, social interaction and customer satisfaction also influence consumer patronage (105), which can create a competitive advantage to avoid losing customers to supermarkets that can offer lower prices and a wider selection (106). Moll Brandão et al. (29) emphasize that the social component of corner stores is more influential in terms of purchase decisions and patronage than a good product range and shop design, achieved through a good relationship with store owners and other customers, which creates a pleasant shopping experience.

The presence of competitors and other retail establishments in the vicinity also affects the demand for specific products and the decision on product offerings and store locations (107, 108). Then, surveys and purchase inventories can help corner stores understand the frequency of shopping and motivations for choosing a particular store and what products are frequently bought (7). Moreover, purchasing behavior can be influenced by other factors. One of them is product placement, which in the case of children’s purchases that are often impulsive and influenced by the prominent placement of sugary and salty snacks near the counter (43). Other factors are the offer of coupons and discounts to attract price-sensitive customers and stimulate purchases (23), in-store cooking demonstrations to educate customers on preparing healthy meals, which can also increase sales of fresh produce (65), provide excellent customer service to enhance the shopping experience and encourage repeat visits (11), regularly update store displays, improve cleanliness, and enhance the store’s exterior to create a welcoming environment (13, 109).

### 4.4 Business models and economic impact

Corner stores are crucial for the social, economic and cultural vitality of neighborhoods (110), impacting the neighborhood economy, job market, and quality of life of residents (111), and being resilient to economic and institutional challenges from urban decline, competing against large supermarkets and wholesale retailers, and the emergence of large discount stores (112). The business models and economic roles of neighborhood stores respond to these challenges through location strategies (113), stocking practices (114), community engagement strategies, customer demographics and surrounding competition (115), adaptation to local demand and economic constraints (116), and customer service and relationship marketing (28, 117).

Corner store customers often prioritizes location and the shopping environment, making them to be chosen based on their convenience and the overall experience they offer, which includes factors like cleanliness, layout, ambiance, positive customer service, availability of culturally appropriate items, and quality fresh fruit (11, 113). In this regard, placement of stock near the front of corner stores increased purchase of produce, which incentivize corner stores to stock and prominently display good-quality produce to their consume (118). The, the physical arrangement of products and their assortment within the store can significantly influence consumer purchase decisions, so corner stores may adjust their layout to optimize product visibility and accessibility, thereby increasing sales (119). Likewise, the financial viability of carrying products also determines the supply of goods to consumers, so the main sources of profit in corner stores tend to be tobacco, hot food items, beer and soft drinks (4, 120).

To satisfy local demand and economic constraints, corner stores can adopt a model where they act as satellites of larger chains, stocking a selected mix of products specifically designed to meet the needs of low-income communities and ensuring that the product range is relevant and accessible (121). It implies the use of dynamic sourcing models and marketing to cater to the specific needs of their community, offering products at affordable prices and reaching community members with limited economic resources (122). Similarly, in rural areas, store owners are adapting by selling products that meet local demand, while focusing on customer service and product quality (123). However, many corner stores rely on cash-and-carry practices for fresh produce, which can limit the variety and quantity of products available, representing a need for good logistics management (114). Then, collaborations between suppliers and corner stores can increase access to products in low-income neighborhoods, representing a new model to increase access to community (124). In this sense, long-term relationships between store owners and distributors can increase influence over promotions, product placement, stocking decisions and point-of-sale advertising, thereby reducing perceived financial risk or customer dissatisfaction (125).

In addition to providing goods, corner stores can also provide services that act as mechanisms for financial inclusion. In Chile, for example, correspondent banking services have been introduced in corner stores, allowing people to access a range of banking and payment services (126). Other consumer-focused services include loyalty programs through mobile apps, which offer rewards and discounts to frequent customers and provide valuable data for targeted marketing and promotions (127). Other technologies, such as solar dehumidification systems, help to reduce energy costs in corner stores, enabling the use of refrigeration to increase the supply and shelf life of fresh and perishable products (128). In this way, the use of new technologies such as smartphones, mobile payments and solar panels increases the adaptability and agility of corner stores to compete with supermarket chains (129).

One of the main management problems of corner stores is that they do not keep electronic records, making it difficult to implement programs to improve sales, such as the introduction of point-of-sale (POS) systems to track sales. Through devices such as tablets, corner stores can implement POS systems, facilitating their uptake due to their cultural acceptability, operability and perceived sustainability by shop owners (130). Considering that the inventory management process in corner stores is done manually based on experience, as the data generated is ignored or even no sales records are kept, it is necessary to develop new strategies and tools that use data analytics techniques. This is expected to predict and refine product quantities in order to maximize profits for the store owners (131). However, for digital transformation strategies to be successful, training, investment and incentives for store owners must be met (132). By overcoming these barriers, it is possible for corner stores to improve their management models by benefiting from statistical models for decision support, such as supervised learning models and statistical exploratory analysis methods such as *t*-test, logistic regression, discrimination analysis, decision tree, random forest and artificial neural networks (131, 133).

In terms of customer service and relationship marketing, corner stores’ business models emphasize trust, commitment, satisfaction and loyalty to build strong relationships with their customers. These relationships are often rooted in cultural and social interactions that are maintained through regular engagement and community events (28). Customer relationships focus on attracting and retaining customers, building loyalty and long-term engagement within a specific geographic area. To do this, traditional marketing tools such as interacting with the local community at events, local print media publications, distributing advertising flyers to local consumers, sponsoring local events and gift vouchers are effective in engaging local consumers (117, 134).

In this sense, effective digital marketing strategies for corner stores can be supported by digital platforms to share stories that resonate with the local community (117), and mobile commerce to deliver timely and relevant promotional strategies to customers in their immediate vicinity through geofencing (135). Corner stores can leverage social media to enhance customer communication, build brand awareness, and increase profits at a lower cost, as customers can spread positive word-of-mouth to more potential consumers online (136). In this way, corner stores, through their business models, can become engines of economic development, generating profitability for the members who create and manage them, developing skills in the marketing of goods and services for mass consumption, creating a reference business model to encourage others to become shop owners, and absorbing the cultural, social and economic elements of the success of those who maintain them (25).

## 5 Discussion

This systematic review consolidates insights from multiple studies to provide a comprehensive understanding of the role of corner stores as key urban institutions. The analysis reveals that corner stores are more than just retail spaces; they act as essential hubs for public health initiatives, socio-economic stability, consumer behavior dynamics, and small business resilience. While prior studies have largely focused on specific aspects of corner stores such as their influence on food access, business viability, or social function, this review integrates these perspectives to highlight their interconnected roles in shaping urban communities.

### 5.1 Public health and nutrition implications

One of the most extensively studied aspects of corner stores is their impact on public health and nutrition. The literature consistently finds that these stores primarily offer energy-dense, low-nutrient foods, which contribute to unhealthy dietary patterns, especially in low-income communities. However, interventions aimed at increasing access to healthy foods through stocking adjustments, promotional strategies, and community engagement have shown moderate success in shifting consumer purchasing behavior. Nonetheless, challenges remain, including consumer preferences for unhealthy foods, financial constraints faced by store owners, and infrastructural limitations that hinder the stocking of fresh produce. Future research should explore the long-term sustainability of such interventions and the factors that influence their effectiveness across different socio-economic contexts.

### 5.2 Socio-cultural and economic role of corner stores

Beyond their function as food retailers, corner stores serve as vital community spaces, fostering social interactions and reinforcing neighborhood cohesion. Many studies highlight that these stores provide informal financial support through credit extensions and personalized service, which is particularly important in low-income and immigrant communities. Moreover, corner stores contribute to local economies by generating employment and supporting small-scale entrepreneurship. Despite these positive contributions, corner stores also face reputational and operational challenges, such as being perceived as sites that promote unhealthy food choices or contribute to neighborhood inequities. Addressing these concerns requires a balance between economic sustainability and community-oriented business practices.

### 5.3 Consumer behavior and purchasing patterns

Purchasing decisions at corner stores are influenced by a combination of convenience, price sensitivity, and product availability. Consumers such as children and adolescents frequently purchase sugary beverages, snacks, and processed foods due to their affordability and accessibility. Although some studies suggest that interventions like nutritional labeling, point-of-sale promotions, and pricing incentives can encourage healthier choices, long-term behavior change remains difficult to achieve. Furthermore, the cultural and social dimensions of shopping experiences influence consumer loyalty, with strong personal relationships between shop owners and customers playing a critical role in shaping store patronage.

### 5.4 Business models and economic sustainability

The economic sustainability of corner stores is a recurring theme in the literature, with studies indicating that store owners often rely on high-margin, low-nutrient products for profitability. While some business models integrate healthier food offerings, they often require financial assistance, supplier collaborations, or technological upgrades to be viable. In this regard, emerging trends such as mobile payments, digital marketing, and supply chain innovations present opportunities for improving store efficiency and competitiveness. However, small-scale retailers often lack the resources or expertise to adopt these innovations, highlighting a need for targeted support programs.

This systematic review contributes to the literature by offering a multidisciplinary perspective on corner stores, synthesizing insights from public health, urban economics, consumer behavior, and community development. Unlike individual studies that focus on isolated aspects, this review underscores the interconnected nature of these dimensions, demonstrating that effective interventions require holistic, cross-sectoral approaches. The findings suggest that future research should not only assess the effectiveness of corner store interventions but also consider broader economic and policy implications to enhance their role in sustainable urban development.

## 6 Conclusion

Corner or neighborhood stores, representing small businesses, play a multifaceted role in urban communities, particularly in low-income and underserved areas. The systematic review on corner stores reveals a growing academic interest in their role within urban food environments, public health, community development, economic stability, and social cohesion. Over recent decades, studies have increasingly focused on the intersection of corner stores and public health, as evidenced by the rise in publications across health, nutrition, and social science disciplines. Most research has been published as journal articles, demonstrating the topic’s relevance within peer-reviewed, scholarly journals. The increase in international collaborations also suggests a recognition of corner stores’ relevance beyond the United States, with global perspectives contributing to a more comprehensive understanding of how these stores influence food access and consumer behavior across diverse communities.

This systematic review highlights the multidimensional role of corner stores in urban environments, demonstrating their impact on public health, community dynamics, consumer behavior, and economic sustainability. While these small retailers serve as critical access points for food and essential goods, they also influence dietary habits, local economies, and social cohesion. Research predominantly focuses on public health interventions, particularly efforts to increase access to healthy foods, yet challenges such as consumer preferences, economic pressures, and infrastructural limitations persist. The socio-economic role of corner stores as hubs of community interaction and informal financial support is well-documented, yet their sustainability remains an ongoing concern, especially in the face of competition from larger retailers. Despite emerging business models that integrate healthier food offerings and technology, many store owners lack the financial or logistical capacity to implement these changes. By synthesizing findings from diverse disciplines, this review provides a comprehensive perspective on corner stores, underscoring the need for holistic, cross-sectoral approaches to enhance the sustainability of these stores while addressing public health concerns.

### 6.1 Limitations

This systematic review relied on a title-based search strategy in Scopus and Web of Science, using the terms “corner store” and “neighborhood store” to identify relevant studies. While this approach ensured a high level of specificity, capturing studies where these stores were a central focus, it may have excluded articles that examined corner stores under alternative terminology, such as “bodega,” “convenience store,” “small food retailer,” or “small grocery store.” This limitation is particularly relevant for research focusing on urban areas with large Latino communities, where the term “bodega” is more commonly used.

In addition, by restricting our search to titles only, we prioritized studies that explicitly focused on corner stores but may have missed articles that discussed them within broader food environments or urban retail contexts. A more comprehensive approach, such as searching abstracts and keywords, or including additional databases such as Google Scholar, PubMed or regional repositories, could yield a larger and potentially more diverse dataset. Future research could refine and extend the search strategy by incorporating more inclusive terminology and exploring additional databases, thereby ensuring a more comprehensive representation of studies on corner shops in different geographical and cultural contexts.

### 6.2 Future research

Future research on corner stores should explore several areas to enhance their role in promoting public health, community development, and business sustainability to support urban communities. While many studies assess the short-term effectiveness of nutrition interventions, little is known about long-term consumer behavior change and the sustainability of these programs. Future research should explore how store-level interventions influence dietary habits over time and whether they lead to lasting improvements in health outcomes. Additionally, future research lines could explore whether interventions focused on specific demographics, like youth or elderly populations, show varying levels of effectiveness in driving healthy purchasing behaviors.

Future research must extend beyond health-focused interventions to examine the broader economic, technological, and policy dimensions that shape the role of corner stores in urban sustainability. The viability of corner stores remains underexplored, particularly in the context of rising competition from large retailers and digital commerce. Research should examine how economic pressures shape product offerings and whether business models that integrate healthier foods can be financially viable without external funding.

Future work should also explore the role of digital payment systems, supply chain innovations, and mobile retail solutions in modernizing corner stores, assessing how technological advancements can enhance store efficiency and consumer engagement, especially in low-income and underserved areas. Likewise, existing research is heavily concentrated in high-income countries, particularly the United States, leaving gaps in our understanding of corner stores in the Global South. Comparative studies across different regions can provide insights into how policy frameworks, economic conditions, and cultural factors influence the function of these stores.

While much research examines food purchases in the context of nutrition, less attention has been given to explore how corner stores support cultural identity, particularly within immigrant and minority communities, and how they respond to the evolving needs of these populations. These studies could include the interplay between community values, cultural relevance, consumer loyalty, and purchasing patterns, as well as include the best practices for community involvement.
